# Lung Function Changes are More Common in Marfan Patients Who Need Major Thoracic Surgery

**DOI:** 10.1007/s00408-019-00236-1

**Published:** 2019-05-14

**Authors:** Abigel M. Kolonics-Farkas, Bence Agg, Kalman Benke, Balazs Odler, Aniko Bohacs, Zsuzsanna Kovats, Zoltan Szabolcs, Veronika Müller

**Affiliations:** 1 Department of Pulmonology, Semmelweis University, Budapest, Hungary; 2 Heart and Vascular Centre, Semmelweis University, Budapest, Hungary; 3 Hungarian Marfan Foundation, Budapest, Hungary; 4 Department of Pharmacology and Pharmacotherapy, Semmelweis University, Budapest, Hungary; 5 Clinical Division of Nephrology, Department of Internal Medicine, Medical University of Graz, Graz, Austria

**Keywords:** Marfan syndrome, Musculoskeletal disorder, Lung function testing, Airway obstruction, Thoracic surgery

## Abstract

**Introduction:**

Marfan syndrome is a genetic disorder affecting the connective tissue. Changes in lung tissue might influence respiratory function; however, a detailed respiratory functional assessment according to the need for major thoracic surgery is missing.

**Methods:**

Comprehensive pulmonary examinations were performed in 55 Marfan patients including respiratory symptoms, lung function (LF) testing using European Coal and Steel Community (ECSC) reference values, TL_CO_ and quality of life measurements. Groups included patients who did not need surgery (Mf, *n* = 32) and those who underwent major thoracic surgery (Mf_op_, *n* = 23).

**Results:**

Respiratory symptoms affected 20% of patients. Scoliosis was significantly more frequent in the Mf_op_ group. LF demonstrated in all Marfan patients a tendency towards airway obstruction (FEV1/FVC = 0.77 ± 0.10), more prominent in Mf_op_ patients (0.74 ± 0.08 vs. Mf: 0.80 ± 0.11; *p* = 0.03). Correction of LF values using a standing height modification by arm span (H_corrected_) revealed additional changes in FVC and FEV1. TL_CO_ and quality of life did not differ between groups.

**Conclusions:**

Marfan syndrome is associated with airway obstruction, especially in patients who have undergone major thoracic surgery, indicative of more severe connective tissue malfunction. The use of arm span for height correction is suitable to evaluate LF changes in this special patient group including patients with significant scoliosis.

## Introduction

Marfan syndrome is a systemic, autosomal dominantly inherited connective tissue disorder, first described in 1896 by Antoine Marfan [1, 2]. In 1991, Francesco Ramirez identified the underlying changes in the glycoprotein fibrillin 1, encoded by the *FBN1* gene, located on chromosome 15 at position 15q21.1 [3]. In approximately 25% of cases, a de novo mutation can be observed [4]. Fibrillin 1, a principal component of microfibrils, plays a key role in the formation and protection of the extracellular matrix [5]. Microfibrils support elastin deposition, and are therefore essential components of elastic fibres [6]. The prevalence of Marfan disease is about 0.2 ‰ [7]. Since this condition is the consequence of connective tissue weakness, it has diverse symptoms. To ease the diagnostic process, the main symptoms have been collected to a unified nosology (Ghent criteria, 1996) [7]. In 2010, a revision of the criteria abolished major and minor criteria and emphasised the value of genetic testing [8]. Regarding lung manifestations, little information is available on the effects of connective tissue changes in the respiratory system; only a few pleuropulmonary abnormalities are known. Chest deformities or dissection of the ascending aorta can affect the mechanics of the ventilatory pump. Structural changes to the lungs can lead to apical blebs and bullae or result in spontaneous pneumothorax [9, 10]. Sleep apnoea is also observed as a consequence of the involvement of the upper airways [11].

Lung function (LF) values measured by spirometry and plethysmography are influenced by thoracic structures such as the airways, lung parenchyma, pleura and muscles; thus, functional changes in LF parameters used in routine clinical practice might be influenced by Marfan syndrome [12]. However, the reference values used in patients with the special body measurements characteristic of Marfan syndrome can be misleading, and comparative measures are lacking [13, 14] . In the present study, our aim was to assess changes to the respiratory system in this rare inherited connective tissue disorder using different reference equations.

## Materials and Methods

### Study Subjects

The study had a cross-sectional design. Following a written inquiry, 55 Caucasian patients from the National Marfan Registry (established and supervised by the Hungarian Marfan Foundation) agreed to participate in the study. Patients were diagnosed with Marfan syndrome using the revised Ghent nosology [8] and/or genetic confirmation (Table 3).

### Study Design

Pulmonary examinations were voluntary. After signing the informed consent, a detailed respiratory assessment was carried out in the Department of Pulmonology, Semmelweis University, Budapest, Hungary between the 31 March 2015 and 4 September 2017. Exclusion criteria were age < 16 years old and major thoracic surgery within 6 months before the assessment. Major thoracic surgery was usually prophylactic aortic root surgery [15, 16] or chest wall surgery and spine correction.

Data on respiratory symptoms (dyspnoea, cough, sputum, chest pain), smoking history, sex, age, height, bodyweight, body mass index (BMI) and arm span (cm) were collected. All patients underwent arterialised earlobe blood gas analysis (Cobas b 221, Roche, Budapest, Hungary), chest X-ray and fluoroscopy, laboratory testing and electrocardiography. The 6-minute walk test (6MWT) was performed to measure exercise capacity according to American Thoracic Society (ATS) guidelines [17]. The extent of scoliosis was measured according to the Cobb method [18]. To assess general quality of life (QoL), the QoL Visual Analogue Scale (VAS) was used. To identify patient health-related conditions, the COPD Assessment Test (CAT®, Hungarian version) [19] and modified Medical Research Council Dyspnoea Scale (mMRC) were applied [20].

The study protocol was approved by the Semmelweis University Regional and Institutional Committee of Science and Research Ethics (TUKEB 165/2016) in accordance with the Declaration of Helsinki.

### Lung Function Measurements

LF measurements included forced vital capacity (FVC), forced expiratory volume in the first second (FEV1), FEV1/FVC, forced expiratory flow between 25 and 75% of FVC (FEF25–75), total lung capacity (TLC), residual volume (RV) and functional residual capacity (FRC) by means of electronic spirometer and body plethysmography (PDD-301/s, Piston, Budapest, Hungary) according to the European Respiratory Society and ATS guidelines [12]. Three technically acceptable manoeuvres were performed and the highest value of them was used. Transfer factor (TL_CO_) and coefficient (KL_CO_) of the lung for carbon monoxide were measured with single breath method (PDD-301/s, Piston, Budapest, Hungary). LF variables are expressed as percentage of predicted values.

As baseline reference values, we used the database of the European Coal and Steel Community (ECSC) set by the spirometry manufacturer [21]. ECSC is used in all Hungarian lung function laboratories. ECSC spirometry reference calculations are the following: FVC men: 5.76H - 0.026A - 4.34; FVC women: 4.43H - 0.026A - 2.89 and FEV1 men: 4.30H - 0.029A - 2.49; FEV1 women: 3.95H - 0.025A - 2.69; (H—height, A—age).

Reference equations using measured height (H_measured_), age and gender may be inappropriate in Marfan syndrome patients due to their special skeletal features, especially following thoracic surgery. To overcome these thoracic abnormalities, we used arm span to correct for height (H_corrected_) [22]. For homogeneous Caucasian populations, the following equations are recommended by Parker et al. [23]:

Males: H_corrected_ (m) = 68.74 + 0.63008·Arm span (m) − 0.1019A.

Females: H_corrected_ (m) = 33.14 + 0.79499·Arm span (m).

We recalculated the spirometric values based on H_corrected_ by applying the original ECSC equations. The range of accuracy in the recommendations for forced expiratory manoeuvres FVC and FEV1 is ± 3% of reading or ± 0.050 L, whichever is greater [12].

### Statistical Analysis

Statistical analysis was performed with GraphPad software (Graph Pad Prism 5.0 by Graph Pad Software Inc., San Diego, USA). Data are presented as mean and standard deviation for continuous data and as median and range for categorical data, respectively. Differences between groups for parametric data were compared using Student’s *t* test, while Fisher’s exact test was applied for analysing non-parametric data. Pearson correlation was performed to test connection between the degree of scoliosis and lung function values. In all cases, *p* < 0.05 was considered statistically significant.

## Results

Patient characteristics are summarised in Table 1. The average age was 38.1 ± 13.1 years. Most patient were never smokers. In the Mf_op_ group, patients had undergone major thoracic surgery mainly due to cardiac causes. Height correction resulted in significantly lower values in Mf patients; however, this difference was only observed in men.

**Table 1:** Patient characteristics

	All patients (*n* = 55)	Mf group (*n* = 32)	Mf_op_ group (*n* = 23)	*p* value Mf versus Mf_op_
Age (years)	38.1 ± 13.1			
Men	32.6 ± 11.6	32.4 ± 11.0	33.9 ± 11.1	n.s
Women	40.8 ± 13.2^a^	37.9 ± 10.9	45.1 ± 14.8	n.s
Gender				
Men, *n* (%)	20 (36)	11 (34)	9 (39)	n.s
Women, *n* (%)	35 (64)	21 (66)	14 (61)	n.s
Weight (kg)	71.7 ± 17.5			
Men	79.1 ± 22.2	79.8 ± 20.3	80.4 ± 23.3	n.s
Women	67.1 ± 12.2	68.1 ± 14.5	67.4 ± 8.9	n.s
Height (m)				
(a) Measured	182.3 ± 10.0	183.1 ± 8.5	181 ± 11.8	n.s
(b) Corrected	179.5 ± 7.4^a^	180.4 ± 6.4^a^	177 ± 8.4	n.s
Men				
a. Measured	191.7 ± 7.9	191.6 ± 9.1	191.7 ± 7.3	n.s
b. Corrected	186.3 ± 6.5	187.0 ± 6.6^a^	185.2 ± 6.6	n.s
Women				
a. Measured	176.5 ± 6.2	178.6 ± 3.6	1.73.9 ± 8.3	n.s
b. Corrected	176.0 ± 5.0	177.3 ± 3.2	174.0 ± 6.6	n.s
BMI (kg/m^2^)	21.5 ± 4.5			
Men	21.5 ± 5.7	21.1 ± 4.7	23.0 ± 6.2	n.s
Women	21.5 ± 3.7	21.1 ± 4.4	22.3 ± 2.8	n.s
Arm span (cm)	185.1 ± 9.3			
Men, *n* (%)	191.8 ± 10.2	193.0 ± 10.2	190.3 ± 9.9	n.s
Women, *n* (%)	181.7 ± 6.8	183.3 ± 4.4	179.1 ± 8.7	n.s
Smoking habit				
Never smoker, *n* (%)	40 (73)	25 (78)	15 (65)	n.s
Former smoker, *n* (%)	11 (20)	5 (16)	6 (26)	n.s
Current smoker, *n* (%)	4 (7)	2 (6)	2 (9)	n.s
Major thoracic surgery indication				
Cardiac, *n* (%)	19 (35)	0	19 (35)	Not analysed
Chest or spine deformity, *n* (%)	4 (7)	0	0	

^a^Significant difference compared to the value above

**Fig. 1:**
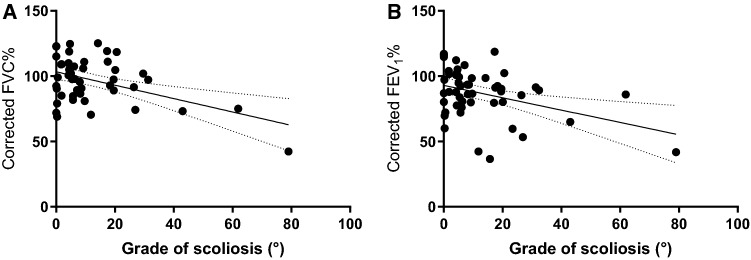
Correlation between extent of scoliosis and height corrected FVC% (**a**) and FEV1% (**b**)

Chest deformities and respiratory symptoms are summarised in Table 2. Significantly more patients suffered from scoliosis in the Mf_op_ group. Significant negative correlation between the extent of scoliosis and FVC% (*r* =  − 0.414, [95% CI − 0.617 to − 0.159], *p* = 0.0023) and FEV1% (*r* =  − 0.401, [95% CI − 0.607 to − 0.144], *p* = 0.003) were noted. Similarly, H_corrected_ FVC% (*r* =  − 0.463, [95% CI − 0.661 to − 0.206], *p* < 0.001) and FEV1% (*r* =  − 0.386, [95% CI − 0.599 to − 0.125], *p* = 0.005) confirmed the association (Fig. 1.).

**Table 2:** Chest deformities and respiratory symptoms in patients with Marfan syndrome

	All patients (*n* = 55)	Mf group (*n* = 32)	Mf_op_ group (*n* = 23)	*p* value Mf versus Mf_op_
Chest deformities				
Pectus carinatum, *n* (%)	24 (48)	12 (38)	12 (52)	n.s
Pectus excavatum, *n* (%)	14 (28)	6 (19)	6 (26)	n.s
Scoliosis, *n* (%)	36 (72)	15 (47)	21 (91)	< 0.01
Asymmetric chest, *n* (%)	19 (38)	11 (34)	8 (35)	n.s
Structural abnormalities of the lung				
Spontaneous pneumothorax, *n* (%)	5 (10)	3 (9)	2 (9)	n.s
Apical blebs and bullae, *n* (%)	4 (8)	3 (9)	1 (4)	n.s
Pleuropulmonary symptoms				
Cough, *n* (%)	11 (20)	5 (16)	6 (26)	< 0.01
Sputum, *n* (%)	5 (9)	1 (3)	4 (17)	n.s
Dyspnoea, *n* (%)	10 (18)	3 (9)	7 (30)	< 0.01
Chest pain, *n* (%)	9 (16)	2 (6)	7 (30)	0.03
Ghent nosology, *n* (%)				
Dilatation of the ascending aorta	38 (69)	20 (61)	18 (78)	n.s
Dissection of the ascending aorta	7 (13)	2 (7)	5 (22)	n.s
Mitral valve prolapse	48 (87)	28 (87)	20 (86)	n.s
Dilatation or dissection of descending aorta	1 (2)	0 (0)	1 (6)	n.s
Reduced upper-to-lower segment ratio	8 (14)	5 (16)	3 (12)	n.s
Increased arm span-to-height ratio	8 (15)	4 (14)	4 (17)	n.s
Wrist sign	47 (85)	29 (90)	18 (79)	n.s
Thumb sign	51 (92)	28 (86)	23 (100)	n.s
Reduced extension at the elbows	5 (9)	1 (3)	4 (18)	n.s
Medial displacement of the medial malleolus causing pes planus	30 (55)	16 (52)	14 (61)	n.s
Heel deformity	8 (15)	5 (17)	3 (12)	n.s
Protrusio acetabulae of any degree	1 (2)	1 (3)	0 (0)	n.s
Joint hypermobility	29 (52)	17 (52)	12 (53)	n.s
Highly arched palate with crowding of teeth	35 (63)	20 (62)	15 (65)	n.s
Facial appearance	35 (63)	19 (59)	16 (68)	n.s
Dolichocephaly	11 (20)	5 (17)	6 (24)	n.s
Enophthalmos	12 (22)	7 (21)	6 (24)	n.s
Downslanting palpebral fissures	9 (17)	7 (21)	3 (12)	n.s
Malar hypoplasia	8 (15)	4 (14)	4 (18)	n.s
Retrognathia	17 (30)	8 (24)	9 (41)	n.s
Ectopia lentis	15 (28)	7 (23)	8 (35)	n.s
Myopia over 3 diopters	29 (52)	16 (50)	13 (56)	n.s
Increased axial length of the globe	3 (6)	2 (7)	1 (6)	n.s
Hypoplastic iris or hypoplastic ciliary muscle causing decreased miosis	1 (2)	0 (3)	0 (0)	n.s
Lumbosacral dural ectasia	4 (7)	4 (10)	0 (0)	n.s
Striae atrophicae (stretch marks)	36 (66)	22 (69)	14 (61)	n.s
Positive family history	32 (58)	18 (56)	14 (60)	n.s
Sex (male)	20 (36)	11 (34)	9 (39)	n.s
Systemic score	8 ± 2	8 ± 2	8 ± 2	n.s
FBN1 mutation identified	40 (73)	21 (62)	19 (84)	n.s

*FBN1* Fibrillin 1

Respiratory symptoms were present in fewer than 20% of patients. Dyspnoea, cough and chest pain were significantly more frequent Mf_op_ patients. Structural changes assessed by chest CT scans of the lungs were scarce.

The LF data using the ECSC reference and H_measured_ are summarised in Table 3. Mf_op_ patients had significantly lower FVC, IVC (inspiratory vital capacity) and TLC as compared to Mf patients. FEV1/FVC was 0.74 ± 0.08 in Mf_op_ and 0.80 ± 0.11 in Mf patients, suggesting an obstructive ventilatory pattern in operated patients. Obstruction severity in Mf_op_, expressed as % predicted FEV1, corresponded to moderate changes. Airway obstruction in Mf_op_ patients was confirmed by significantly decreased FEF25–75 values as compared to Mf patients. Increased RV and FRC, both signs of hyperinflation, were observed in both groups.

**Table 3:** Lung function testing in Mf and Mf_op_ using H_measured_ for the ECSC equations

	All patients (*n* = 55)	Mf group (*n* = 32)	Mf_op_ group (*n* = 23)	*p* value Mf versus Mf_op_
FVC (L)	4.20 ± 1.10	4.53 ± 1.06	3.75 ± 1.02	0.01
FVC (%)	93.38 ± 17.54	97.55 ± 15.66	86.48 ± 18.05	0.02
FEV1 (L)	3.24 ± 0.10	3.60 ± 0.93	2.76 ± 0.79	< 0.01
FEV1 (%)	84.13 ± 18.52	91.06 ± 17.02	75.06 ± 16.69	< 0.01
FEF25–75 (L)	2.96 ± 1.24	3.40 ± 1.20	2.35 ± 0.99	< 0.01
FEF25–75 (%)	71.49 ± 29.50	80.32 ± 31.16	59.40 ± 21.18	0.01
PEF (L)	6.25 ± 1.72	6.56 ± 1.63	5.90 ± 1.81	n.s
PEF (%)	74.25 ± 18.08	77.39 ± 18.77	70.99 ± 16.79	n.s
RV (%)	125.86 ± 30.42	128.45 ± 34.67	124.03 ± 27.01	n.s
FRC (%)	122.70 ± 26.42	120.85 ± 27.66	124.03 ± 25.45	n.s
TLC (L)	5.90 ± 1.26	6.27 ± 1.20	5.41 ± 1.20	0.01
TLC (%)	87.83 ± 14.51	92.97 ± 11.41	82.57 ± 16.33	< 0.01
IVC (L)	4.16 ± 1.08	4.43 ± 1.06	3.80 ± 1.03	0.03
IVC (%)	87.25 ± 16.82	91.27 ± 15.29	82.72 ± 17.82	0.05
FEV1/FVC	0.77 ± 0.10	0.80 ± 0.11	0.74 ± 0.08	0.03
FEV1/IVC	0.80 ± 0.16	0.82 ± 0.12	0.71 ± 0.18	< 0.01
TL_CO_ (mmol/min/kPa)	10.01 ± 2.83	10.74 ± 2.82	9.24 ± 2.68	n.s
TL_CO_ (%)	89.55 ± 18.43	94.64 ± 17.97	85.17 ± 18.02	n.s
KL_CO_ [mmol/min/kPa/L]	1.72 ± 0.32	1.77 ± 0.30	1.68 ± 0.34	n.s
KL_CO_ (%)	80.57 ± 17.11	80.69 ± 19.00	81.50 ± 14.68	n.s
Blood gases				
pH	7.42 ± 0.02	7.41 ± 0.02	7.42 ± 0.01	n.s
pO_2_ (mmHg)	83.28 ± 7.02	83.88 ± 6.24	82.41 ± 8.09	n.s
pCO_2_ (mmHg)	37.42 ± 3.21	37.13 ± 0.02	37.84 ± 3.19	n.s
6MWT				
Distance (m)	566.7 ± 99.06	584.28 ± 92.82	542.22 ± 104.27	n.s.
Heart rate change (1/min)	34.40 ± 12.65	40.03 ± 11.20	26.57 ± 7.43	n.s.
O_2_ saturation change (%)	1.02 ± 8.36	1.53 ± 2.4	0.30 ± 1.36	n.s.
QoL				
VAS (1–100)	78.39 ± 19.67	81.37 ± 18.01	74.16 ± 21.61	n.s
CAT (0–40)^a^	7 (0–22)	7 (0–22)	10 (0–22)	n.s
mMRC (0–4)^a^	0 (0–3)	0 (0–2)	1 (0–3)	n.s

*FVC* forced vital capacity, *FEV1* forced expiratory volume in the first second, *FEF25–75* forced expiratory flow between 25 and 75%, *PEF* peak expiratory flow, *RV* residual volume, *FRC* functional residual capacity, *TLC* total lung capacity

^a^Data expressed as median (range)

Diffusion (TL_CO_ and KL_CO_), blood gases, 6MWT data or QoL were not different between groups (Table 3). CAT® and mMRC showed higher values in the Mf_op_ group with more respiratory symptoms.

Using arm span corrected height, FVC and FEV1% predicted values increased in all patient groups (Table 4.). FEV1 remained in the pathological range in Mfop patients ( < 80% predicted) and stayed significantly lower as compared to Mf group.

**Table 4:** Lung function parameters using ECSC with H_measured_ and H_corrected_ in Marfan patients

	All patients (*n* = 55)	Mf group (*n* = 32)	Mf_op_ group (*n* = 23)	*p* value Mf versus Mf_op_
FVC%				
ECSC H_measured_ (%)	93.38 ± 17.54	97.55 ± 15.66	86.48 ± 18.05	0.02
ECSC H_corrected_ (%)	96.68 ± 18.09	101.99 ± 15.18	88.02 ± 19.15	0.01
FEV1%				
ECSC H_measured_ (%)	84.13 ± 18.52	91.06 ± 17.02	75.06 ± 16.69	< 0.01
ECSC H_corrected_ (%)	86.41 ± 23.49	93.27 ± 16.68	77.25 ± 18.92	< 0.01

## Discussion

Our study is the largest cohort of Marfan patients who were serially assessed for pulmonary involvement. Twenty percent of the patients complained about pulmonary symptoms. Cough, dyspnoea and chest pain were common, affecting a higher proportion of Mf_op_ patients. QoL measures correlated with symptoms.

LF values are usually based on age, sex and standing height, which may be misleading in Marfan syndrome, where the length of the lower limbs contributes disproportionally to height [24]. As height can be corrected by arm span, we used equations to overcome this height measurement bias. This resulted in a significant decrease in the height values of Mf group patients, especially in men, leading to the conclusion that, in many Marfan patients, standard LF reference values are disproportionally high.

In 1960, the ECSC was the first organisation to issue recommendations for the calculation of reference values [25]. The reference values described by the ECSC were based on males working in coal mines and steel works. This was not a representative reference population, and in practice the predicted values were considered to be too high. Although no women had been tested, the ECSC calculated reference values for females at 80% of the values for men [14].

Our data confirmed airway obstruction, mainly affecting the small airways, in all Marfan patients. Similar results were previously observed in a study by Streeten and al [26]. The novelty of our study is the subgrouping according to major thoracic surgery. It is of high clinical importance to ensure appropriate lung function during or following extensive thoracic interventions. As a majority of Mf_op_ patients had scoliosis, it is not surprising that the measured and corrected heights did not differ in these patients. However, height correction revealed abnormal FVC and FEV1 values.

Airway obstruction was moderate in all patients. This change might result from connective tissue malfunction in this young patient population due to their disease. The changes might also reflect incipient emphysema and/or an increased tendency for the airways to collapse [27]. Due to the pathological structure of fibrillin 1, the development of emphysema can be often observed in these patients. Robbesom et al. showed that aberrant fibrillin 1 in the lung is significantly associated with the three most important morphometric parameters of emphysema: alveolar destruction, airspace enlargement and emphysema-related morphological abnormalities; [28] experimental data in mice have confirmed widening of the distal airspaces in Marfan syndrome [29]. As described by Hogg et al., small airways are the main site of obstruction in lungs affected by emphysema [30]. It is suspected that areas with trapped air develop emphysema over time [31]. Combined with the increased tendency of the small airways to collapse in Marfan syndrome [27], it can be assumed that, due to connective tissue malfunction, air trapping starts in the small airways, which later might convert into emphysema.

Six of our patients (10,9%) were diagnosed with asthma, 5 of them well controlled (Mf *n* = 3, Mfop *n* = 2) without obstructive ventilatory changes at the time of assessment. One patient awaiting cardiac surgery presented with mixed ventilatory pattern. No further patients had clinical signs of asthma. The extent of scoliosis showed significant negative correlation with FVC% and FEV1%, pointing towards restrictive changes due to thorax abnormalities.

Our data suggest that LF evaluation in patients with atypical anthropometrical features can be difficult. The equations applied in LF testing might give different results and it might be beneficial to reassess results in those who have unusual physical features.

## Conclusion

This study performed a complex respiratory functional assessment of a large cohort of Marfan patients, confirming previous data showing mild obstructive ventilatory disorder. The need for major thoracic surgery was associated with more respiratory symptoms, more severe functional changes and worse QoL. Height correction revealed decreased FVC and FEV1 values in Mf_op_ patients more in line with their clinical symptoms. Small airway obstruction in our patients indicates that particular attention is needed in the follow-up of respiratory status. One weakness of our study is that the reversibility of airway obstruction in Mf_op_ patients was not investigated in the absence of clinical symptoms of asthma. The extent of scoliosis showed significant negative correlation with FVC% and FEV1% suggestive of restrictive changes due to thoracic deformities. Longitudinal data will be needed to evaluate changes of airway function in Marfan syndrome. In daily clinical practice, more attention should be placed on pulmonary involvement and LF assessments when planning or after major thoracic surgery in Marfan patients.

## References

[1] Marfan A (1896) Un cas de déformation congénitale des quatre membres, plus prononcée aux extrémités, caractérisée par l’allongement des os avec un certain degré d’amincissement Impr Maretheux

[2] Judge DP, Dietz HC (2005). Marfan’s syndrome. Lancet.

[3] Colovati ME, da Silva LR, Takeno SS, Mancini TI, Dutra AR, Guilherme RS, de Mello CB, Melaragno MI, Perez AB (2012). Marfan syndrome with a complex chromosomal rearrangement including deletion of the FBN1 gene. Mol Cytogenet.

[4] Dyhdalo K, Farver C (2011). Pulmonary histologic changes in Marfan syndrome: a case series and literature review. Am J Clin Pathol.

[5] Seyama Y, Hayashi M, Usami E, Yamashita S (1992). Change in elastin structure in human aortic connective tissue diseases. Amino Acids.

[6] Kielty CM (2017). Fell-Muir Lecture: Fibrillin microfibrils: structural tensometers of elastic tissues?. Int J Exp Pathol.

[7] De Paepe A, Devereux RB, Dietz HC, Hennekam RCM, Pyeritz RE (1996). Revised diagnostic criteria for the Marfan syndrome. Am J Med Genet.

[8] Loeys BL, Dietz HC, Braverman AC, Callewaert BL, De Backer J, Devereux RB, Hilhorst-Hofstee Y, Jondeau G, Faivre L, Milewicz DM (2010). The revised Ghent nosology for the Marfan syndrome. J Med Genet.

[9] Hao W, Fang Y, Lai H, Shen Y, Wang H, Lin M, Tan L (2017). Marfan syndrome with pneumothorax: case report and review of literatures. J Thorac Dis.

[10] Corsico AG, Grosso A, Tripon B, Albicini F, Gini E, Mazzetta A, Di Vincenzo EM, Agnesi ME, Tsana Tegomo E, Ronzoni V (2014). Pulmonary involvement in patients with Marfan Syndrome. Panminerva Med.

[11] Neuville M, Jondeau G, Crestani B, Taillé C (2015). Respiratory manifestations of Marfan’s syndrome. Rev Mal Respir.

[12] Miller MR (2005). Standardisation of spirometry. Eur Respir J.

[13] Quanjer PH, Stanojevic S, Cole TJ, Baur X, Hall GL, Culver BH, Enright PL, Hankinson JL, Ip MSM, Zheng J (2012). Multi-ethnic reference values for spirometry for the 3–95-yr age range: the global lung function 2012 equations. Eur Respir J.

[14] Quanjer PH, Stanojevic S, Stocks J, Cole TJ GLI-2012 reference values for spirometry GLI-2012 All-Age Multi-Ethnic Reference Values for Spirometry Advantages Consequences GLI-2012 reference values for spirometry Interpretation of spirometric data

[15] Pearson GD, Devereux R, Loeys B, Maslen C, Milewicz D, Pyeritz R, Ramirez F, Rifkin D, Sakai L, Svensson L (2008). Report of the national heart, lung, and blood institute and national marfan foundation working group on research in marfan syndrome and related disorders. Circulation.

[16] Benke K, Ágg B, Szabó L, Szilveszter B, Odler B, Pólos M, Cao C, Maurovich-Horvat P, Radovits T, Merkely B (2016). Bentall procedure: quarter century of clinical experiences of a single surgeon. J Cardiothorac Surg.

[17] ATS Statement (2012) 10.1164/AJRCCM.166.1.AT1102

[18] Goldberg CJ, Kaliszer M, Moore DP, Fogarty EE, Dowling FE (2001). Surface topography, Cobb angles, and cosmetic change in scoliosis. Spine (Phila Pa 1976).

[19] Jones PW, Harding G, Berry P, Wiklund I, Chen W-H, Leidy NK (2009). Development and first validation of the COPD Assessment Test. Eur Respir J.

[20] Bestall JC, Paul EA, Garrod R, Garnham R, Jones PW, Wedzicha JA (1999). Usefulness of the Medical Research Council (MRC) dyspnoea scale as a measure of disability in patients with chronic obstructive pulmonary disease. Thorax.

[21] Piston User Manual. [date unknown]

[22] Measuring arm span. [date unknown]

[23] Parker JM, Dillard TA, Phillips YY (1996). Arm span-height relationships in patients referred for spirometry. Am J Respir Crit Care Med.

[24] Pyeritz RE, McKusick VA (1979). The Marfan syndrome: diagnosis and management. N Engl J Med.

[25] Jouasset D (1960). Standardization of respiratory function tests in countries of the European coal and steel region. Poumon Coeur.

[26] Streeten E (1987). Pulmonary function in the Marfan syndrome. Chest.

[27] Giske L, Stanghelle JK, Rand-Hendrikssen S, Strøm V, Wilhelmsen J-E, Røe C (2003). Pulmonary function, working capacity and strength in young adults with Marfan syndrome. J Rehabil Med.

[28] Robbesom AA, Koenders MM, Smits NC, Hafmans T, Versteeg EM, Bulten J, Veerkamp JH, Dekhuijzen R, Van Kuppevelt TH (2008). Aberrant fibrillin-1 expression in early emphysematous human lung: a proposed predisposition for emphysema. Mod Pathol.

[29] Uriarte JJ, Meirelles T, Del Blanco DG, Nonaka PN, Campillo N, Sarri E, Navajas D, Egea G, Farré R (2016). Early impairment of lung mechanics in a murine model of Marfan syndrome. PLoS ONE.

[30] Hogg JC, Macklem PT, Thurlbeck WM (1967). The resistance of small airways in normal and diseased human lungs. Aspen Emphysema Conf.

[31] Hogg JC, Paré PD, Hackett T-L (2017). The contribution of small airway obstruction to the pathogenesis of chronic obstructive pulmonary disease. Physiol Rev.

